# An original phylogenetic approach identified mitochondrial haplogroup T1a1 as inversely associated with breast cancer risk in *BRCA2* mutation carriers

**DOI:** 10.1186/s13058-015-0567-2

**Published:** 2015-04-25

**Authors:** Sophie Blein, Claire Bardel, Vincent Danjean, Lesley McGuffog, Sue Healey, Daniel Barrowdale, Andrew Lee, Joe Dennis, Karoline B Kuchenbaecker, Penny Soucy, Mary Beth Terry, Wendy K Chung, David E Goldgar, Saundra S Buys, Breast Cancer Family Registry, Ramunas Janavicius, Laima Tihomirova, Nadine Tung, Cecilia M Dorfling, Elizabeth J van Rensburg, Susan L Neuhausen, Yuan Chun Ding, Anne-Marie Gerdes, Bent Ejlertsen, Finn C Nielsen, Thomas VO Hansen, Ana Osorio, Javier Benitez, Raquel Andrés Conejero, Ena Segota, Jeffrey N Weitzel, Margo Thelander, Paolo Peterlongo, Paolo Radice, Valeria Pensotti, Riccardo Dolcetti, Bernardo Bonanni, Bernard Peissel, Daniela Zaffaroni, Giulietta Scuvera, Siranoush Manoukian, Liliana Varesco, Gabriele L Capone, Laura Papi, Laura Ottini, Drakoulis Yannoukakos, Irene Konstantopoulou, Judy Garber, Ute Hamann, Alan Donaldson, Angela Brady, Carole Brewer, Claire Foo, D Gareth Evans, Debra Frost, Diana Eccles, EMBRACE, Fiona Douglas, Jackie Cook, Julian Adlard, Julian Barwell, Lisa Walker, Louise Izatt, Lucy E Side, M John Kennedy, Marc Tischkowitz, Mark T Rogers, Mary E Porteous, Patrick J Morrison, Radka Platte, Ros Eeles, Rosemarie Davidson, Shirley Hodgson, Trevor Cole, Andrew K Godwin, Claudine Isaacs, Kathleen Claes, Kim De Leeneer, Alfons Meindl, Andrea Gehrig, Barbara Wappenschmidt, Christian Sutter, Christoph Engel, Dieter Niederacher, Doris Steinemann, Hansjoerg Plendl, Karin Kast, Kerstin Rhiem, Nina Ditsch, Norbert Arnold, Raymonda Varon-Mateeva, Rita K Schmutzler, Sabine Preisler-Adams, Nadja Bogdanova Markov, Shan Wang-Gohrke, Antoine de Pauw, Cédrick Lefol, Christine Lasset, Dominique Leroux, Etienne Rouleau, Francesca Damiola, GEMO Study Collaborators, Hélène Dreyfus, Laure Barjhoux, Lisa Golmard, Nancy Uhrhammer, Valérie Bonadona, Valérie Sornin, Yves-Jean Bignon, Jonathan Carter, Linda Van Le, Marion Piedmonte, Paul A DiSilvestro, Miguel de la Hoya, Trinidad Caldes, Heli Nevanlinna, Kristiina Aittomäki, Agnes Jager, Ans MW van den Ouweland, Carolien M Kets, Cora M Aalfs, Flora E van Leeuwen, Frans BL Hogervorst, Hanne EJ Meijers-Heijboer, HEBON, Jan C Oosterwijk, Kees EP van Roozendaal, Matti A Rookus, Peter Devilee, Rob B van der Luijt, Edith Olah, Orland Diez, Alex Teulé, Conxi Lazaro, Ignacio Blanco, Jesús Del Valle, Anna Jakubowska, Grzegorz Sukiennicki, Jacek Gronwald, Jan Lubinski, Katarzyna Durda, Katarzyna Jaworska-Bieniek, Bjarni A Agnarsson, Christine Maugard, Alberto Amadori, Marco Montagna, Manuel R Teixeira, Amanda B Spurdle, William Foulkes, Curtis Olswold, Noralane M Lindor, Vernon S Pankratz, Csilla I Szabo, Anne Lincoln, Lauren Jacobs, Marina Corines, Mark Robson, Joseph Vijai, Andreas Berger, Anneliese Fink-Retter, Christian F Singer, Christine Rappaport, Daphne Geschwantler Kaulich, Georg Pfeiler, Muy-Kheng Tea, Mark H Greene, Phuong L Mai, Gad Rennert, Evgeny N Imyanitov, Anna Marie Mulligan, Gord Glendon, Irene L Andrulis, Sandrine Tchatchou, Amanda Ewart Toland, Inge Sokilde Pedersen, Mads Thomassen, Torben A Kruse, Uffe Birk Jensen, Maria A Caligo, Eitan Friedman, Jamal Zidan, Yael Laitman, Annika Lindblom, Beatrice Melin, Brita Arver, Niklas Loman, Richard Rosenquist, Olufunmilayo I Olopade, Robert L Nussbaum, Susan J Ramus, Katherine L Nathanson, Susan M Domchek, Timothy R Rebbeck, Banu K Arun, Gillian Mitchell, Beth Y Karlan, Jenny Lester, Sandra Orsulic, Dominique Stoppa-Lyonnet, Gilles Thomas, Jacques Simard, Fergus J Couch, Kenneth Offit, Douglas F Easton, Georgia Chenevix-Trench, Antonis C Antoniou, Sylvie Mazoyer, Catherine M Phelan, Olga M Sinilnikova, David G Cox

**Affiliations:** INSERM U1052, CNRS UMR5286, Université Lyon 1, Centre de Recherche en Cancérologie de Lyon, Lyon, France; Université de Lyon, 69000 Lyon, France; Université Lyon 1, 69100 Villeurbanne, France; UMR CNRS 5558, Laboratoire de Biométrie et Biologie Évolutive (LBBE), “Biométrie et Biologie Évolutive”, Université Claude Bernard Lyon 1, Bâtiment Grégor Mendel, 43 boulevard du 11 novembre 1918, 69622 Villeurbanne, cedex France; Université Grenoble Alpes, UMR 5217, Laboratoire d’Informatique de Grenoble (LIG), équipe-projet Multi-programmation et Ordonnancement sur ressources pour les Applications Interactives de Simulation (MOAIS), 38041 Grenoble, France; INRIA Rhône-Alpes, équipe-projet MOAIS, 38334 Saint Ismier, Cedex France; Centre for Cancer Genetic Epidemiology, Department of Public Health and Primary Care, University of Cambridge, Cambridge, UK; Department of Genetics and Computational Biology, QIMR Berghofer, Brisbane, Australia; Centre de recherche du Centre hospitalier universitaire de Québec, Laval University, Charlesbourg, PQ Canada; Department of Epidemiology, Mailman School of Public Health, Columbia University, New York, NY USA; Department of Pediatrics, Columbia University College of Physicians and Surgeons, New York, NY USA; Department of Medicine, Columbia University College of Physicians and Surgeons, New York, NY USA; Department of Dermatology, University of Utah School of Medicine, Salt Lake City, UT USA; Department of Internal Medicine, Huntsman Cancer Institute, University of Utah School of Medicine, Salt Lake City, UT USA; Department of Epidemiology, Cancer Prevention Institute of California, 2201 Walnut Avenue, Suite 300, Fremont, CA 94538 USA; Hematology, Oncology and Transfusion Medicine Center, Vilnius University Hospital Santariskiu Clinics, Vilnius, Lithuania; Department of Molecular and Regenerative Medicine, Centre for Innovative Medicine, State Research Institute, Vilnius, Lithuania; Latvian Biomedical Research and Study Centre, Rātsupītes iela 1, Rīga, LV-1067 Latvia; Division of Hematology Oncology, Beth Israel Deaconess Medical Center, 330 Brookline Avenue, Shapiro 9, Boston, MA 02215-5400 USA; Department of Genetics, University of Pretoria, Private Bag X20, Hatfield, 0028 Pretoria, South Africa; Department of Population Sciences, Beckman Research Institute, City of Hope, 1500 East Duarte Road, Duarte, CA 91010 USA; Department of Clinical Genetics, Rigshospitalet, Copenhagen University Hospital, Copenhagen, Denmark; Department of Oncology, Rigshospitalet, Copenhagen University Hospital, Copenhagen, Denmark; Center for Genomic Medicine, Rigshospitalet, Copenhagen University Hospital, Copenhagen, Denmark; Human Genetics Group, Spanish National Cancer Research Center (CNIO), Madrid, Spain; Center for Biomedical Network Research on Rare Diseases (CIBERER), Madrid, Spain; Medical Oncology Service, Hospital Clínico Universitario Lozano Blesa, Avenida San Juan Bosco, 15, 50009 Zaragoza, Spain; Holy Cross Hospital, Michael and Dianne Bienes Comprehensive Cancer Center, Fort Lauderdale, FL USA; Division of Clinical Cancer Genetics, City of Hope (for the Clinical Cancer Genetics Community Research Network), City of Hope, 1500 East Duarte Road, Duarte, CA 91010 USA; John Muir Medical Center, Walnut Creek, CA, USA; c/o Clinical Cancer Genetics Community Research Network, City of Hope, 1500 East Duarte Road, Duarte, CA 91010 USA; Istituto FIRC di Oncologia Molecolare (IFOM), Via Adamello 16, 20139 Milan, Italy; Unit of Molecular Bases of Genetic Risk and Genetic Testing, Department of Preventive and Predictive Medicine, Istituto di Ricovero e Cura a Carattere Scientifico (IRCCS), Istituto Nazionale dei Tumori (INT), Via Venezian 1, 20133 Milan, Italy; Cogentech Cancer Genetic Test Laboratory, Via Adamello 16, 20139 Milan, Italy; Cancer Bioimmunotherapy Unit, Centro di Riferimento Oncologico (CRO), Via Franco Gallini 2, 33081 Aviano, Italy; Division of Cancer Prevention and Genetics, Istituto Europeo di Oncologia, Via Ripamonti 435, 20141 Milan, Italy; Unit of Medical Genetics, Department of Preventive and Predictive Medicine, Istituto di Ricovero e Cura a Carattere Scientifico (IRCCS), Istituto Nazionale dei Tumori (INT), Via Venezian 1, 20133 Milan, Italy; Unit of Hereditary Cancer, Department of Epidemiology, Prevention and Special Functions, Istituto di Ricovero e Cura a Carattere Scientifico (IRCCS), Azienda Ospedaliera Universitaria “San Martino” di Genova, IST Istituto Nazionale per la Ricerca sul Cancro, Largo Rosanna Benzi, 10, 16132 Genoa, Italy; FiorGen Foundation for Pharmacogenomics, Via Luigi Sacconi 6, 50019 Sesto Fiorentino, Italy; Unit of Medical Genetics, Department of Biomedical, Experimental and Clinical Sciences, University of Florence, Florence, Italy; Department of Molecular Medicine, Sapienza University, Rome, Italy; Department of Medical Oncology, Papageorgiou Hospital, Aristotle University of Thessaloniki School of Medicine, Thessaloniki, Greece; Molecular Diagnostics Laboratory, INRASTES, National Centre for Scientific Research “Demokritos”, Aghia Paraskevi Attikis, Athens, Greece; Dana-Farber Cancer Institute, 450 Brookline Avenue, Boston, MA 02215 USA; Molecular Genetics of Breast Cancer, Deutsches Krebsforschungszentrum (DKFZ), Heidelberg, Germany; Clinical Genetics Department, St Michael’s Hospital, Southwell Street, Bristol, BS2 8EG UK; North West Thames Regional Genetics Service, Kennedy-Galton Centre, Harrow, UK; Department of Clinical Genetics, Royal Devon & Exeter Hospital, Barrack Road, Exeter, EX2 5DW UK; Merseyside and Cheshire Clinical Genetics Service, Liverpool Women’s NHS Foundation Trust, Crown Street, Liverpool, Merseyside L8 7SS UK; Genetic Medicine, Manchester Academic Health Sciences Centre, Central Manchester University Hospitals NHS Foundation Trust, Manchester, UK; Centre for Cancer Genetic Epidemiology, Department of Public Health and Primary Care, Strangeways Research Laboratory, University of Cambridge, Worts Causeway, Cambridge, CB1 8RN UK; Faculty of Medicine, University of Southampton, Southampton University Hospitals NHS Trust, Mailpoint 801, South Academic Block, PAH/G/MP105, Tremona Road, Southampton, SO16 6YD UK; Institute of Human Genetics, Northern Genetic Service, International Centre for Life, Newcastle upon Tyne Hospitals NHS Trust, Central Parkway, Newcastle upon Tyne, NE1 4EP UK; Sheffield Clinical Genetics Service, Sheffield Children’s Hospital, Sheffield, UK; Yorkshire Regional Genetics Service, Leeds Teaching Hospitals NHS Trust, Old Medical School, Leeds General Infirmary, Leeds, LS1 3EX UK; Leicestershire Clinical Genetics Service, Department of Clinical Genetics, Leicester Royal Infirmary, University Hospitals of Leicester NHS Trust, Leicester, LE1 5WW UK; Oxford Regional Genetics Service, Churchill Hospital, Old Road, Headington, Oxford, OX3 7LE UK; Clinical Genetics Service, Guy’s and St Thomas’ NHS Foundation Trust, 7th floor, Borough Wing, Guy’s Hospital, Great Maze Pond, London, SE1 9RT UK; North East Thames Regional Genetics Service, Great Ormond Street Hospital for Children NHS Trust, Barclay House, 37, Queen Square, London, WC1N 3BH UK; Academic Unit of Clinical and Molecular Oncology, Trinity College Dublin, College Green, Dublin 2, Ireland; Medical Oncology Service, St James’s Hospital, James’s Street, Dublin 8, Ireland; Department of Clinical Genetics, East Anglian Regional Genetics Service, Addenbrooke’s Hospital, Level 6, Addenbrooke’s Treatment Centre, Cambridge University Hospitals NHS Foundation Trust, Hills Road, Cambridge, CB2 0QQ UK; All Wales Medical Genetics Services, University Hospital of Wales, Heath Park, Cardiff, CF14 4XW UK; South East Scotland Regional Genetic Service, Western General Hospital, David Brock Building, Crewe Road South, Edinburgh, EH4 2XU UK; Centre for Cancer Research & Cell Biology, School of Medicine, Dentistry and Biomedical Sciences, Queen’s University Belfast, 97 Lisburn Road, Belfast, BT9 7AE UK; Department of Medical Genetics, Belfast Health and Social Care Trust, Belfast City Hospital, Lisburn Road, Belfast, BT9 7AB UK; Oncogenetics Team, The Institute of Cancer Research and Royal Marsden NHS Foundation Trust, 123 Old Brompton Road, London, SW7 3RP UK; Ferguson-Smith Centre for Clinical Genetics, Yorkhill Hospitals, Block 4, Glasgow, G3 8SJ UK; South West Thames Regional Genetics Service, Department of Medical Genetics, St George’s University of London, Cranmer Terrace, London, SW17 0RE UK; West Midlands Regional Genetics Service, Birmingham Women’s Hospital Healthcare NHS Trust, Mindelsohn Way, Edgbaston, Birmingham, B15 2TG UK; Department of Pathology & Laboratory Medicine, University of Kansas Medical Center, 3901 Rainbow Boulevard, Kansas City, KS 66160 USA; Lombardi Comprehensive Cancer Center, MedStar Georgetown University Hospital, 3800 Reservoir Road NW, Washington, DC 20057 USA; Center for Medical Genetics, Ghent University Hospital, De Pintelaan 185, 9000 Ghent, Belgium; Division of Tumor Genetics, Department of Gynaecology and Obstetrics, University Hospital Klinikum Rechts der Isar, Technische Universität München, Ismaninger Strasse 22, 81675 Munich, Germany; Center of Familial Breast and Ovarian Cancer, Department of Medical Genetics, Institut für Humangenetik, Biozentrum, Universität Würzburg, Am Hubland, 97074 Würzburg, Germany; Center for Hereditary Breast and Ovarian Cancer, Medical Faculty, Center for Integrated Oncology (CIO) Cancer Center Cologne, University Hospital Cologne, Cologne, Germany; Center for Molecular Medicine Cologne (CMMC), University of Cologne, Robert-Koch-Strasse 21, 50931 Cologne, Germany; Department of Human Genetics, Institute of Human Genetics, University Hospital Heidelberg, Heidelberg, Germany; Institute for Medical Informatics, Statistics and Epidemiology, Medical Faculty, University of Leipzig, Leipzig, Germany; Department of Gynaecology and Obstetrics, University Hospital Düsseldorf, Heinrich-Heine University Düsseldorf, Moorenstrasse 5, 40225 Düsseldorf, Germany; Institute of Cell and Molecular Pathology, Centre for Pathology and Forensic and Genetic Medicine, Hannover Medical School, Carl-Neuberg-Strasse 1, 30625 Hannover, Germany; Institute of Human Genetics, University Medical Center Schleswig-Holstein, Campus Kiel, Arnold-Heller-Strasse 3, D-24105 Kiel, Germany; Department of Gynecology and Obstetrics, University Hospital Carl Gustav Carus of Dresden, Technical University Dresden, Dresden, Germany; Department of Gynecology and Obstetrics, University Medical Center Schleswig-Holstein, Campus Kiel, Arnold-Heller-Strasse 3, D-24105 Kiel, Germany; Institute of Medical Genetics and Human Genetics, Campus Virchow-Klinikum, Charité Berlin – Universtitätsmedizin Berlin, Augustenburger Platz 1, 13353 Berlin, Germany; German Consortium of Hereditary Breast and Ovarian Cancer (GC-HBOC), Cologne, Germany; Institute of Human Genetics, University Hospital Münster, Vesaliusweg 12-14, 48149 Münster, Germany; Department of Gynecology and Obstetrics, University Hospital Ulm, Ulm, Germany; Department of Tumour Biology, Institut Curie, 26 rue d’Ulm 75248, Paris, cedex 05 France; Unité de Prévention et d’Épidémiologie Génétique, Centre Léon Bérard, 28 rue Laenned, 69008 Lyon, France; Génétique Clinique, Centre Hospitalier Universitaire de Grenoble, CS 10217, 38043, Grenoble, cedex 9 France; Institut Albert Bonniot – Inserm U823, Université Joseph Fourier, Rond-point de la Chantourne, 38706 La Tronche, France; Laboratoire d’Oncogénétique, Hôpital René Huguenin, Institut Curie, 35 rue Dailly, 92210 Saint-Cloud, France; Département d’Oncogénétique, Centre Jean Perrin, Université de Clermont-Ferrand, 58 rue Montalembert, BP 392, 63011 Clermont-Ferrand, France; Gynaecological Oncology, Sydney Cancer Centre, Royal Prince Alfred Hospital and University of Sydney, Missenden Road, Camperdown, NSW 2050 Australia; Gynecologic Oncology Group, Department of OB-GYN, University of North Carolina at Chapel Hill, 103B Physicians’ Office Building, CB# 7572, Chapel Hill, NC 27599-7572 USA; Gynecologic Oncology Group Statistical and Data Center, Roswell Park Cancer Institute, Elm and Carlton Streets, Buffalo, NY 14263-0001 USA; Women & Infants Hospital, 1 Blackstone Place, Providence, RI 02905 USA; Molecular Oncology Laboratory, Health Research Institute of the San Carlos Clinical Hospital (IdISSC), 28040 Madrid, Spain; Department of Obstetrics and Gynecology, University of Helsinki and Helsinki University Central Hospital, Biomedicum Helsinki, PO Box 700, 00029 Helsinki, Finland; Department of Clinical Genetics, Helsinki University Central Hospital, Biomedicum Helsinki 1, Haartmaninkatu 8, 00290 Helsinki, Finland; Department of Medical Oncology, Family Cancer Clinic, Erasmus University Medical Center, PO Box 2040, 3000 CA Rotterdam, the Netherlands; Department of Clinical Genetics, Family Cancer Clinic, Erasmus University Medical Center, Rotterdam, the Netherlands; Department of Human Genetics, Radboud University Nijmegen Medical Centre, Nijmegen, the Netherlands; Department of Clinical Genetics, Academic Medical Center, Amsterdam, the Netherlands; Department of Epidemiology, Netherlands Cancer Institute, Amsterdam, the Netherlands; Family Cancer Clinic, Netherlands Cancer Institute, Amsterdam, the Netherlands; Department of Clinical Genetics, VU University Medical Center Amsterdam, De Boelelaan 1118, 1081 HV Amsterdam, the Netherlands; Department of Genetics, University Medical Center, Groningen University, Groningen, the Netherlands; Department of Clinical Genetics, Maastricht University Medical Center, Maastricht, the Netherlands; Department of Human Genetics, Center for Human and Clinical Genetics, Leiden University Medical Center, S4-P PO Box 9600, 2300 RC Leiden, the Netherlands; Department of Pathology, Leiden University Medical Center, PO Box 9600, 2300 RC L1Q Leiden, the Netherlands; Department of Medical Genetics, University Medical Center Utrecht, Utrecht, the Netherlands; Department of Molecular Genetics, National Institute of Oncology, Ráth György u 7-9, PO Box 1525 Budapest PF 21, 1122 Budapest, Hungary; Oncogenetics Group, Vall d’Hebron Institute of Oncology (VHIO), University Hospital Vall d’Hebron, Vall d’Hebron Research Institute (VHIR) and Universitat Autònoma de Barcelona, Passeig de la Vall d’Hebron 119, 08035 Barcelona, Spain; Genetic Counseling Unit, Hereditary Cancer Program, Institut d’Investigació Biomèdica de Bellvitge (IDIBELL)-Catalan Institute of Oncology, Hospital Duran i Reynals, 3a planta - Gran Via de l’Hospitalet, 199, 08908 Hospitalet de Llobregat Barcelona, Spain; Molecular Diagnostic Unit, Hereditary Cancer Program, Institut d’Investigació Biomèdica de Bellvitge (IDIBELL)-Catalan Institute of Oncology, Hospital Duran i Reynals, 3a planta - Gran Via de l’Hospitalet, 199, 08908 Hospitalet de Llobregat Barcelona, Spain; Department of Genetics and Pathomorphology, Faculty of Medicine and Dentistry, Pomeranian Medical University, al Powstancow Wlkp 72, 70-111 Szczecin, Poland; Landspítali National University Hospital of Iceland and Faculty of Medicine, School of Health Sciences, University of Iceland School of Medicine, Sæmundargötu 2, 101 Reykjavik, Iceland; Laboratoire de diagnostic génétique et Service d’Onco-hématologie, Les Hopitaux Universitaire de Strasbourg, Nouvel Hôpital Civil, 1 place de l’Hôpital, BP 426, 67091 Strasbourg, France; Department of Surgical Sciences, Oncology and Gastroenterology, Padua University, Clinical Surgery II, via Giustiniani 2, 35124 Padua, Italy; Immunology and Molecular Oncology Unit, Istituto Oncologico Veneto (IOV) – Istituto di Ricovero e Cura a Carattere Scientifico (IRCCS), via Gattamelata 64, 35128 Padua, Italy; Department of Genetics, Portuguese Oncology Institute (IPO-PORTO), Edifício dos Laboratórios, piso 6, 4200-072 Porto, Portugal; Instituto de Ciências Biomédicas Abel Salazar (ICBAS), Instituto de Ciências Biomédicas Abel Salazar da Universidade do Porto, Rua de Jorge Viterbo Ferreira 228, 4050-313 Porto, Portugal; Program in Cancer Genetics, Departments of Human Genetics and Oncology, McGill University, 546 Pine Avenue West, Montreal, QC J2W 1S6 Canada; Department of Health Sciences Research, Mayo Clinic, 200 First Street SW, Rochester, MN 55905 USA; Department of Health Sciences Research, Mayo Clinic, 13400 East Shea Boulevard, Scottsdale, AZ 85259 USA; National Human Genome Research Institute, National Institutes of Health, Building 31, Room 4B09, 31 Center Drive, MSC 2152, 9000 Rockville Pike, Bethesda, MD 20892-2152 USA; Clinical Genetics Service, Department of Medicine, Memorial Sloan Kettering Cancer Center, 1275 York Avenue, New York, NY 10065 USA; Clinical Genetics Research Laboratory, Memorial Sloan Kettering Cancer Center, 1275 York Avenue, New York, NY 10065 USA; Department of Obstetrics and Gynecology, Comprehensive Cancer Center Vienna, Medical University of Vienna, Universitätsklinik für Frauenheilkunde, AKH – Wien, Währinger Gürtel 18-20, 1090 Vienna, Austria; Clinical Genetics Branch, Division of Cancer Epidemiology and Genetics, National Cancer Institute, National Institutes of Health, Bethesda, MD USA; National Israeli Cancer Control Center and Department of Community Medicine and Epidemiology, Clalit Health Services Carmel Medical Center, 34361 Haifa, Israel; Ruth and Bruce Rappaport Faculty of Medicine, Technion – Israel Institute of Technology, 2 Horev Street, 34362 Haifa, Israel; NN Petrov Institute of Oncology, 68 Leningradskaya Street, Pesochny, 197758 St Petersburg Russia; Department of Laboratory Medicine and Pathobiology, University of Toronto, Medical Sciences Building, 6th Floor, 1 King’s College Circle, Toronto, ON M5S 1A8 Canada; Keenan Research Centre, Li Ka Shing Knowledge Institute, St Michael’s Hospital, 209 Victoria Street, Toronto, ON M5B 1T8 Canada; Ontario Cancer Genetics Network, Cancer Care Ontario, 620 University Avenue, Toronto, ON M5G 2L7 Canada; Lunenfeld-Tanenbaum Research Institute, Mount Sinai Hospital Joseph and Wolf Lebovic Health Complex, 600 University Avenue, Toronto, ON M5G 1X5 Canada; Department of Molecular Genetics, University of Toronto, Medical Science Building, Room 4386, 1 King’s College Circle, Toronto, ON M5S 1A8 Canada; Department of Human Cancer Genetics, 1093 Biomedical Research Tower, 460 West 12th Avenue, Columbus, OH 43210 USA; Department of Internal Medicine, The Ohio State University Wexner Medical Center, North Doan Tower, 395 West 12th Avenue, Columbus, OH 43210 USA; Department of Molecular Virology, Immunology and Medical Genetics, The Ohio State University Wexner Medical Center, 1093 Biomedical Research Tower, 460 West 12th Avenue, Columbus, OH 43210 USA; The Ohio State University Comprehensive Cancer Center – Arthur G James Cancer Hospital and Richard J Solove Research Institute (OSUCCC – James), 460 West 10th Avenue, Columbus, OH 43210 USA; Section of Molecular Diagnostics, Department of Biochemistry, Aalborg University Hospital, Hobrovej 18, 9000 Aalborg, Denmark; Department of Clinical Genetics, Odense University Hospital, Soenderboulevard 29, 5000 Odense C, Denmark; Department of Clinical Genetics, Aarhus University Hospital, Brendstrupgårdsvej 21 C, 8200 Aarhus N, Denmark; Laboratorio di Genetica Oncologica, Divisione di Anatomia Patologica e di Diagnostica Molecolare ed Ultrastrutturale, Azienda Ospedaliero Universitaria Pisana – Ospedale S Chiara, via Roma 67, 56126 Pisa, Italy; Sheba Laboratory of Molecular Genetics, The Danek Gertner Institute of Human Genetics, Sheba Medical Center, Tel Hashomer, Ramat Gan, 52621 Tel Aviv, Israel; Institute of Oncology, Rivka Ziv Medical Center, Maimonides, 13100 Safed, Israel; Department of Cancer Genetics, Karolinska University Hospital, Solna L8:02, SE-171 76 Stockholm, Sweden; Oncology, Department of Radiation Sciences, Umeå University, SE-901 87 Umeå, Sweden; Department of Oncology-Pathology, Karolinska University Hospital, K7, Ärftlighetsmottagningen, Radiumhemmet, 171 76 Stockholm, Sweden; Division of Oncology and Pathology, Department of Clinical Sciences, Lund University Hospital, Barngatan 2B, SE-221 85 Lund, Sweden; Department of Immunology, Genetics and Pathology, Rudbeck Laboratory, Uppsala University, 751 85 Uppsala, Sweden; Center for Clinical Cancer Genetics and Global Health, The University of Chicago, 5841 South Maryland Avenue, Chicago, IL 60637 USA; Department of Medicine and Genetics, University of California, San Francisco, CA USA; Department of Preventive Medicine, Keck School of Medicine, University of Southern California, 1441 Eastlake Avenue, Norris Comprehensive Cancer Center, NOR-4435, Los Angeles, CA 90089-9175 USA; Department of Medicine, Abramson Cancer Center, Perelman School of Medicine, University of Pennsylvania, 3535 Market Street, Suite 750, Philadelphia, PA 19104-3309 USA; Department of Epidemiology and Biostatistics, Abramson Cancer Center, Perelman School of Medicine, University of Pennsylvania, 3535 Market Street, Suite 750, Philadelphia, PA 19104-3309 USA; Division of Cancer Medicine, Department of Breast Medical Oncology, The University of Texas MD Anderson Cancer Center, Unit 1354, PO Box 301439, Houston, TX 77230-1439 USA; Sir Peter MacCallum Department of Oncology, Familial Cancer Centre, Peter MacCallum Cancer Centre, level 3, 10 St Andrews Place, East Melbourne, VIC 3002 Australia; Sir Peter MacCallum Department of Oncology, The University of Melbourne, Level 5, 161 Barry Street, Parkville, 3010 VIC Australia; Women’s Cancer Program, Samuel Oschin Comprehensive Cancer Institute, Cedars-Sinai Medical Center, 8700 Beverly Boulevard, Los Angeles, CA 90048 USA; Service de génétique oncologique, Institut Curie, Inserm U830, 26 rue d’Ulm, 75248 Paris, France; Faculté de Médecine, Université Paris Descartes, Sorbonne Paris Cité, 15 rue de l’école de médecine, 75006 Paris, France; Génétique médicale, Faculté de Médecine Lyon Est, Université Claude Bernard Lyon 1, 8 avenue Rockefeller, 69373 Lyon, Cedex 08 France; Institut National du Cancer (INCa), La Fondation Synergie Lyon Cancer, Centre Léon Bérard, 28 rue Laënnec, 69008 Lyon, Cedex 08 France; Department of Laboratory Medicine and Pathology, Mayo Clinic, 200 First Street SW, Rochester, MN 55905 USA; Department of Cancer Epidemiology, Moffitt Cancer Center, 12902 Magnolia Drive, Tampa, FL 33612 USA; Unité Mixte de Génétique Constitutionnelle des Cancers Fréquents, Hospices Civils de Lyon – Centre Léon Bérard, 69373 Lyon, Cedex 08 France; Clinical Cancer Genetics Community Research Network, City of Hope, 1500 East Duarte Road, Duarte, CA 91010 USA

## Abstract

**Introduction:**

Individuals carrying pathogenic mutations in the *BRCA1* and *BRCA2* genes have a high lifetime risk of breast cancer. *BRCA1* and *BRCA2* are involved in DNA double-strand break repair, DNA alterations that can be caused by exposure to reactive oxygen species, a main source of which are mitochondria. Mitochondrial genome variations affect electron transport chain efficiency and reactive oxygen species production. Individuals with different mitochondrial haplogroups differ in their metabolism and sensitivity to oxidative stress. Variability in mitochondrial genetic background can alter reactive oxygen species production, leading to cancer risk. In the present study, we tested the hypothesis that mitochondrial haplogroups modify breast cancer risk in *BRCA1/2* mutation carriers.

**Methods:**

We genotyped 22,214 (11,421 affected, 10,793 unaffected) mutation carriers belonging to the Consortium of Investigators of Modifiers of *BRCA1/2* for 129 mitochondrial polymorphisms using the iCOGS array. Haplogroup inference and association detection were performed using a phylogenetic approach. ALTree was applied to explore the reference mitochondrial evolutionary tree and detect subclades enriched in affected or unaffected individuals.

**Results:**

We discovered that subclade T1a1 was depleted in affected *BRCA2* mutation carriers compared with the rest of clade T (hazard ratio (HR) = 0.55; 95% confidence interval (CI), 0.34 to 0.88; *P* = 0.01). Compared with the most frequent haplogroup in the general population (that is, H and T clades), the T1a1 haplogroup has a HR of 0.62 (95% CI, 0.40 to 0.95; *P* = 0.03). We also identified three potential susceptibility loci, including G13708A/rs28359178, which has demonstrated an inverse association with familial breast cancer risk.

**Conclusions:**

This study illustrates how original approaches such as the phylogeny-based method we used can empower classical molecular epidemiological studies aimed at identifying association or risk modification effects.

**Electronic supplementary material:**

The online version of this article (doi:10.1186/s13058-015-0567-2) contains supplementary material, which is available to authorized users.

## Introduction

Breast cancer is a multifactorial disease with genetic, lifestyle and environmental susceptibility factors. Approximately 15% to 20% of the familial aggregation of breast cancer is accounted for by mutations in high-penetrance susceptibility genes [1-3], such as *BRCA1* and *BRCA2*. Pathogenic mutations in *BRCA1* and *BRCA2* confer lifetime breast cancer risk of 60% to 85% [4,5] and 40% to 85% [4,5], respectively. Other genomic variations (for example, in genes encoding proteins interacting with *BRCA1* and *BRCA2*) have been identified as modifiers of breast cancer risk and increase or decrease the risk initially conferred by *BRCA1* or *BRCA2* mutation [6].

*BRCA1* and *BRCA2* are involved in DNA repair mechanisms, including double-strand break (DSB) repair by homologous recombination [7,8]. DSBs are considered to be among the most deleterious forms of DNA damage because the integrity of both DNA strands is compromised simultaneously. These breaks can lead to genomic instability resulting in translocations, deletions, duplications or mutations when not correctly repaired [9]. Reactive oxygen species (ROS) are one of the main causes of DSBs, along with exposure to ionizing radiation, various chemical agents and ultraviolet light [10].

ROS are naturally occurring chemical derivatives of metabolism. Elevated levels of ROS and downregulation of ROS scavengers and/or antioxidant enzymes can lead to oxidative stress, which is associated with a number of human diseases, including various cancers [11]. The electron transport chain process, which takes place in the mitochondria, generates the majority of ROS in human cells. Variations in the mitochondrial genome have been shown to be associated with metabolic phenotypes and oxidative stress markers [12]. Mitochondrial dysfunction recently was shown to promote breast cancer cell migration and invasion through the accumulation of a transcription factor, hypoxia-inducible factor 1α, via increased production of ROS [13].

Human mitochondrial DNA (mtDNA) has undergone a large number of mutations that have segregated during evolution. Those changes are now used to define mitochondrial haplogroups. Some of these changes slightly modify metabolic performance and energy production; thus, not all haplogroups have identical metabolic capacities [14]. It has been hypothesized that the geographic distribution of mitochondrial haplogroups results from selection of metabolic capacities driven mainly by adaptation to climate and nutrition [15,16].

Mitochondrial haplogroups have been associated with diverse multifactorial diseases, such as Alzheimer’s disease [17], hypertrophic cardiomyopathy [18], retinal diseases [19] or age-related macular degeneration [20]. Variations in mtDNA have also been linked to several types of cancer, such as gastric cancer [21] or renal cell carcinoma [22]. Interestingly, variations in mtDNA have been linked to several types of female cancers, including endometrial [23], ovarian [24] and breast cancer [25,26]. A recent study underlined the possibility that mtDNA might be involved in the pathogenic and molecular mechanisms of familial breast cancer [27].

The Collaborative Oncological Gene-environment Study [28] (COGS) is a European project designed to improve understanding of genetic susceptibility to breast, ovarian and prostate cancer. This project involves several consortia: the Breast Cancer Association Consortium (BCAC) [29], the Ovarian Cancer Association Consortium [30], the Prostate Cancer Association Group to Investigate Cancer Associated Alterations in the Genome (PRACTICAL) [31] and the Consortium of Investigators of Modifiers of *BRCA1/2* (CIMBA) [32]*.* CIMBA is a collaborative group of researchers working on genetic modifiers of cancer risk in *BRCA1* and *BRCA2* mutation carriers. As part of the COGS project, more than 200,000 single-nucleotide polymorphisms (SNPs) were genotyped for *BRCA1* and *BRCA2* female mutation carriers on the iCOGS chip, including 129 mitochondrial polymorphisms. The iCOGS chip is a custom Illumina™ Infinium genotyping array (Illumina, San Diego, CA, USA) designed to test, in a cost-effective manner, genetic variants related to breast, ovarian and prostate cancers.

In this study, we explored mitochondrial haplogroups as potential modifiers of breast cancer risk in women carrying pathogenic *BRCA1* or *BRCA2* mutations. Our study includes females diagnosed with breast cancer and unaffected carriers belonging to CIMBA. We used an original analytic phylogenetics-based approach implemented in a homemade algorithm and in the program ALTree [33,34] to infer haplogroups and to detect associations between haplogroups and breast cancer risk.

## Methods

### Ethics statement

A signed informed written consent form was obtained from all participants. All contributing studies involved in CIMBA received approvals from the institutional review committees at their host institutions. Ethical committees that approved access to the data analyzed in this study are listed in Additional file 1.

### *BRCA1* and *BRCA2* mutation carriers

Final analyses included 7,432 breast cancer cases and 7,104 unaffected *BRCA1* mutation carriers, as well as 3,989 invasive breast cancer and 3,689 unaffected *BRCA2* mutation carriers, all belonging to CIMBA. Supplementary specifications regarding inclusion profiles and studies belonging to CIMBA are available in the reports by Couch *et al.* [35] and Gaudet *et al.* [36]. All analyses were conducted separately on CIMBA *BRCA1* and *BRCA2* mutation carriers (abbreviated *pop1* and *pop2*, respectively). Eligible female carriers were aged 18 years or older and had a pathogenic mutation in *BRCA1* and/or *BRCA2*. Women with both *BRCA1* and *BRCA2* mutations were included in downstream analyses. Data were available for year of birth, age at study recruitment, age at cancer diagnosis, *BRCA1* and *BRCA2* mutation description and self-reported ethnicity. Women with ovarian cancer history were not excluded from analyses, and they represented 15% and 7% of *BRCA1* and *BRCA2* mutation carriers, respectively. Information regarding mastectomy was incomplete and was therefore not used as an inclusion or exclusion parameter.

### Genotyping and quality filtering

Genotyping was conducted using the iCOGS custom Illumina Infinium array. Data from this array are available to the scientific community upon request. Please see [37] for more information. Genotypes were called using Illumina’s proprietary GenCall algorithm. Genotyping and quality filtering were described previously [35,36]. Initially, 129 mitochondrial SNPs were genotyped for both *BRCA1* and *BRCA2* mutation carriers. SNPs fulfilling the following criteria were excluded from downstream analyses: monoallelic SNPs (minor allele frequency = 0), SNPs with more than 5% data missing, annotated as triallelic, or having probes cross-matching with the nuclear genome. Heterozygous genotypes were removed from analyses, and we further filtered out SNPs having more than 5% of heterozygous calls to limit the potential for heteroplasmy affecting our results. We also did not retain SNPs representing private mutations. These mutations are rare, often restricted to a few families, and not sufficiently prevalent in the general population to be included in the reference mitochondrial evolutionary tree (see below). This last step of filtration yielded 93 and 92 SNPs for the *pop1* and *pop2* analyses, respectively (see Additional file 2). Only individuals with fully defined haplotypes (that is, non-missing genotypes for the 93 and 92 SNPs selected for *pop1* and *pop2*, respectively) were included in downstream analyses (14,536 and 7,678 individuals, respectively).

### Mitochondrial genome evolution and haplogroup definition

Analyses were based on the theoretical reconstructed phylogenetic tree of the mitochondrial genome (mtTree) known as PhyloTree [38] (v.15). The mtTree is rooted by the Reconstructed Sapiens Reference Sequence (RSRS). RSRS has been identified as the most likely candidate to root the mtTree by refining human mitochondrial phylogeny by parsimony [39]. Each haplogroup in mtTree is defined by the set of mtDNA SNPs that have segregated in RSRS until today in the mitochondrial genome. Each haplogroup is fully characterized by the 16,569-bp sequence resulting from the application of all the substitutions that are encoded by the corresponding SNPs in the RSRS sequence.

### Haplogroups imputation

**Figure 1:**
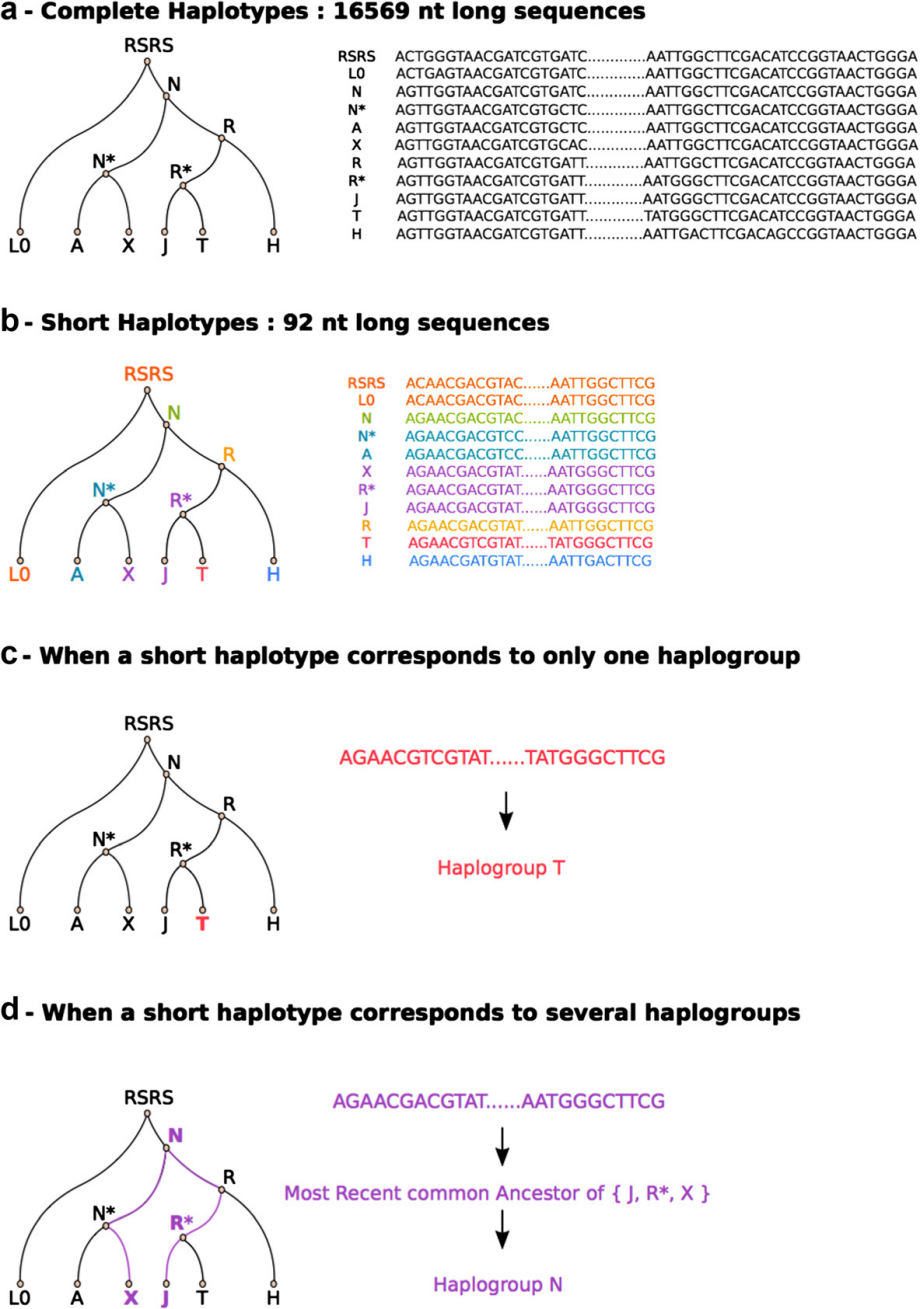
Simplified representation of the phylogenic method used to infer haplogroups. **(a)** Full-length haplotypic sequences are reconstructed at each node of the reference tree. **(b)** Haplotypes are then restricted to available loci. Sequences of the same color are identical. **(c)** Unique short haplotypes are matched directly with the corresponding haplogroup. **(d)** Sequences that match with several haplogroups are associated with their most recent common ancestor haplogroup. RSRS, Reconstructed Sapiens Reference Sequence.

The phylogenetic approach used to infer haplogroups is described in Figure 1. Mitochondrial genome sequences can be reconstructed at each node of mtTree, given the substitutions that have segregated in RSRS. Each haplogroup therefore has a corresponding full-length mitochondrial sequence. However, the full-length mitochondrial sequence is not available in the data, because the iCOGS platform captured only 93 and 92 SNPs for *pop1* and *pop2*, respectively. Thus, for each of the 7,864 nodes of the phylogenetic tree, the corresponding short haplotype (that is, the full-length sequence restricted to available loci) was defined. Some of the short haplotypes are unique, and they can be matched with their corresponding haplogroup directly. However, most of the time, given the small number of SNPs analyzed, several haplogroups correspond to the same short haplotype. Consequently, a unique haplogroup cannot confidently be assigned to each short haplotype. Therefore, each short haplotype was assigned the most recent common ancestor of all the haplogroups that share the same short haplotype. Once this matching was done, short haplotypes were reconstructed in the same way for each individual in our dataset and were assigned the corresponding haplogroup. The accuracy of the method used was assessed by application to a set of 630 mtDNA sequences of known European and Caucasian haplogroups (see Additional file 3).

### Association detection

This phylogenetic approach is based on the identification of subclades in the reference phylogenetic tree of the mitochondrial genome differentially enriched for cases and unaffected controls compared with neighboring subclades. We used ALTree [33,34] to perform association testing. ALTree—standing for Association detection and Localization of susceptibility sites using haplotype phylogenetic Trees—is an algorithm used to perform nested homogeneity tests to compare distributions of affected and unaffected individuals in the different clades of a given phylogenetic tree. The objective is to detect if some clades of a phylogenetic tree are more or less enriched in affected or unaffected individuals compared with the rest of the tree. There are as many tests performed as there are levels in the phylogenetic tree. The *P*-value at each level of the tree is obtained by a permutation procedure in which 1,000 permutations are performed. Individual labels (“affected” or “unaffected”) are permutated 1,000 times to see to what extent the observed distribution of affected or unaffected is different from a random distribution. A procedure to correct for multiple testing adapted to nested tests [40] is implemented in ALTree. The objective of ALTree is to detect an enrichment difference at the level of the whole tree. To conserve computational time and resources, only the most significant *P*-value obtained for all tests performed on one tree is corrected.

### Handling genetic dependency

ALTree is used to perform homogeneity tests to detect differences in enrichment or depletion of affected or unaffected individuals between clades in the phylogenetic tree. This kind of test can be performed only on independent data. However, because some individuals in the CIMBA dataset belong to the same family, we constructed datasets with genetically independent data by randomly selecting one individual from among all those belonging to the same family and sharing the same short haplotype. To take into account the full variability of our data, we resampled 1,000 times. The results of the analysis pipeline are obtained for each resampling independently and then averaged over the 1,000 resamplings to obtain final results.

### Character reconstruction at ancestral nodes

Before the ALTree localization algorithm was launched, ancestral sequences were reconstructed at each internal tree node; that is, short haplotypes were inferred with maximum likelihood at all nodes that were not leaves. We used the software PAML [41] to perform the reconstruction at ancestral nodes using a maximum likelihood method. The phylogeny model used was the general time-reversible model (either GTR or REV).

### Localization of susceptibility sites

ALTree also includes an algorithm used to identify which sites are the most likely ones to be involved in the association detected. For each short haplotype observed, the ALTree add-on *altree-add-S* adds to the short haplotype sequence a supplementary character called *S*, which represents the disease status associated with this short haplotype. Are individuals carrying this short haplotype more often affected or unaffected? *S* is calculated based on the affected and unaffected counts, the relative proportion of affected and unaffected in the whole dataset, and sensibility parameter *ε. ε* was set to its default value, which is 1. After *S* character computation, haplotypes including character *S* are reconstructed at ancestral nodes. Susceptibility site localization is achieved with ALTree by computing a correlated evolution index calculated between each change of each site and the changes of the character *S* in the two possible directions of change. The sites whose evolution are the most correlated with the character *S* are the most likely susceptibility sites.

### Selected subclades

The analyses were carried out on the full evolutionary tree. However, the more haplogroups there are at each level, the less statistical power homogeneity tests have. Therefore, analyses were also applied to subclades extracted from the tree. Subclades were defined using counts of individuals in each haplogroup of the clade to maximize statistical power. The chosen subclades and corresponding affected and unaffected counts are presented in Table 2.

### Statistical analysis

We quantified the effect associated with enrichment discovered by applying ALTree by building a weighted Cox regression in which the outcome variable is the status (affected or non-affected) and the explicative variable is the inferred haplogroup. Analyses were stratified by country. Data were restricted to the clades of interest. The uncertainty in haplogroup inference was not taken into account in the model. The weighting method used takes into account breast cancer incidence rate as a function of age [42] and the gene containing the observed pathogenic mutation (that is, *BRCA1* or *BRCA2*). Familial dependency was handled by using a robust sandwich estimate of variance (R package *survival*, *cluster()* function).

## Results

### Haplogroup imputation

In Additional file 4, absolute and relative frequencies are recapitulated for each haplogroup imputed in *BRCA1* and *BRCA2* mutation carriers. For *BRCA1* mutation carriers, we reconstructed 489 distinct short haplotypes of 93 loci from the genotypes data. Only 162 of those 489 short haplotypes matched theoretical haplotypes reconstructed in the reference mitochondrial evolutionary tree. These 162 haplotypes represented 13,315 of 14,536 individuals. Thus, 91.6% of *BRCA1* mutation carriers were successfully assigned a haplogroup. For *BRCA2* mutation carriers, we reconstructed 350 distinct short haplotypes of 92 loci from our genotype data. Only 139 of those 350 short haplotypes matched theoretical haplotypes reconstructed in the reference mitochondrial evolutionary tree. These 139 haplotypes represented 6,996 of 7,678 individuals. Thus, 91.1% of *BRCA2* mutation carriers were successfully assigned a haplogroup. Because more *BRCA1* than *BRCA2* mutation carriers were genotyped (14,536 vs. 7,678 individuals), we logically observed more distinct haplotypes in *pop1* than in *pop2* (489 vs. 350 haplotypes)*.*

The accuracy of the main haplogroup inference method used was estimated at 82% and reached 100% for haplogroups I, J, K, T, U, W and X. Given the set of SNPs we disposed of, our method has difficulty differentiating between H and V haplogroups (see Additional file 3).

### Association results

For both populations of *BRCA1* or *BRCA2* mutation carriers, as well as for the full tree as for all selected subclades (see Table 1), we extracted the mean corrected *P*-values for association testing over all resamplings performed (see Table 2). The only corrected *P*-value that remained significant was that obtained for subclade T (abbreviated T*) in the population of individuals of *BRCA2* mutation carriers (*P* = 0.04).

**Table 1:** **Counts of participants in selected subclades**

**Subclade**	***BRCA1*** **mutation carriers**	***BRCA2*** **mutation carriers**
U8	1,458	863
T	1,243	651
J	1,270	630
J1	1,043	513
H	3,706	1,967
H1	582	337
U5	868	458
X1′2′3	221	103
K1a	608	364

**Table 2:** **Mean corrected** ***P*** **-values for association testing with ALTree**

**Subclade**	***pop1*** **corrected** ***P*** **-value**	***pop2*** **corrected** ***P*** **-value**
Full	0.830	0.681
U8	0.146	0.626
T	0.285	**0.040**
J	0.718	0.112
J1	0.621	0.150
H	0.747	0.930
H1	0.268	0.804
U5	0.829	0.747
X1′2 ′3	0.416	0.629
K1a	0.170	0.162

^a^ *pop1*, *BRCA1* mutation carrier; *pop2*, *BRCA2* mutation carrier. Bold indicates a significant *P*-value.

**Figure 2:**
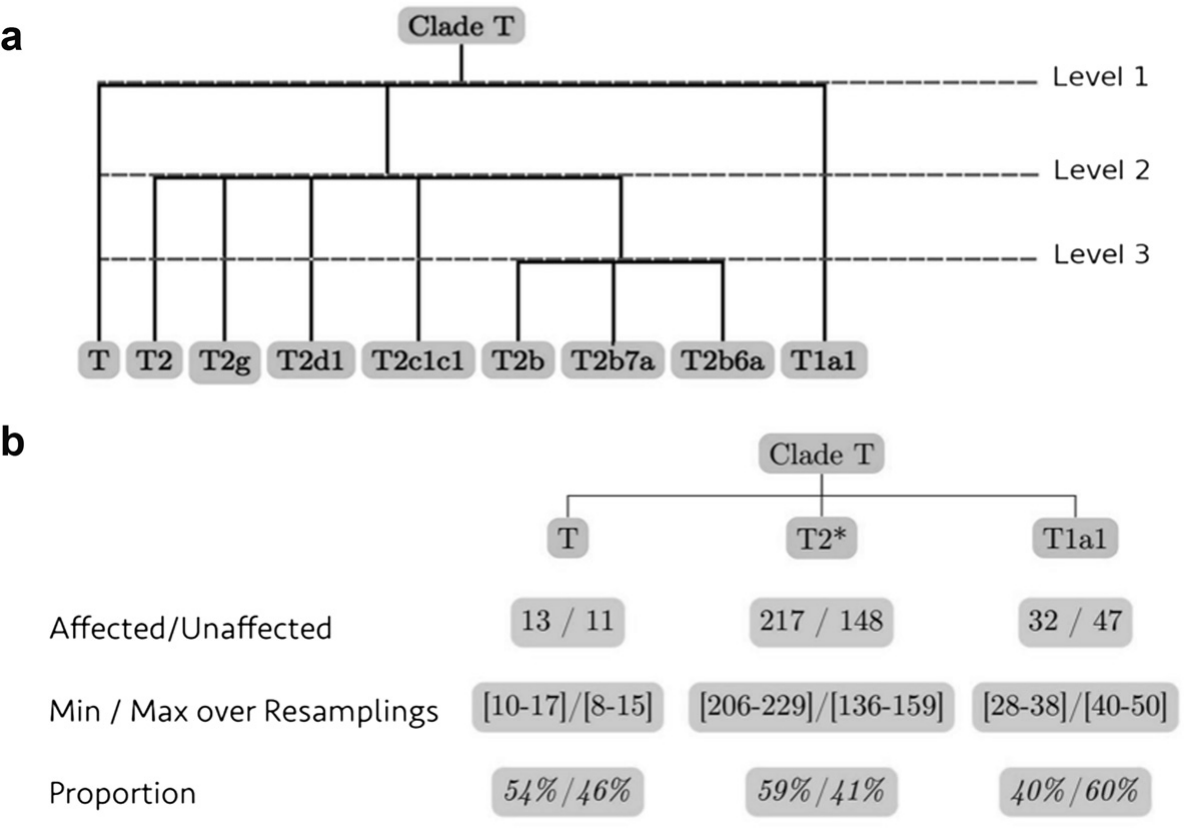
Phylogenetic tree of subclade T tested for association with ALTree. **(a)** Phylogenetic tree of subclade T with all observed haplogroups. A homogeneity test is performed at each level of the tree. **(b)** First level of the phylogenetic tree of subclade T. Averaged counts, ranges and proportions of affected and unaffected observed in resamplings are indicated below each subclade. T2* represents the entire T2 subclade.

The phylogenetic tree of subclade T (see Figure 2a) contains only three levels; thus, only three tests were performed within this clade. Raw *P*-values were examined to determine at which level of the tree ALTree detects a difference of enrichment in affected or unaffected individuals (see Table 3). Only the *P*-value associated with the test performed at the first level of the tree is significant. We looked more closely at the mean frequencies of affected and unaffected individuals in the tree at this level (see Figure 2b). In the T1a1 subclade, the mean count of affected and unaffected are 32 and 47, respectively. In the T2* subclade, we observed, on average, 217 and 148 affected and unaffected individuals, respectively, whereas in the T subclade, we observed, on average, 13 and 11 affected and unaffected individuals, respectively. The ranges observed for each of these values over the 1,000 resamplings are represented in Figure 2b. On the basis of these observations, we conclude that subclade T1a1 is depleted in affected carriers compared with the neighboring subclades T and T2.

**Table 3:** **Non-corrected** ***P*** **-values by level of phylogenetic tree for subclade T in** ***BRCA2*** **mutation carriers**

**Level**	**Degrees of freedom**	**Mean of non-corrected** ***P*** **-value**
1	2	0.02141039
2	6	0.14355900
3	8	0.22249700

### Localization results

We performed a localization analysis with ALTree. The correlated evolution index for all non-monomorphic sites observed in short haplotype sequences of subclade T are displayed in Additional file 5. The higher the correlated evolution index, the more likely it is that corresponding sites will be involved in the observed association. Three short haplotype sites numbered 44, 57 and 72 and corresponding to SNPs T988C, G11812A/rs4154217 and G13708A/rs28359178, respectively, clearly distinguish themselves, with correlation index values of 0.390, 0.324 and 0.318, respectively, whereas the correlation index values of all other sites ranged from −0.270 to −0.101. Table 4 shows the details for these three loci.

**Table 4:** **Description of loci identified as potential susceptibility sites by ALTree** ^**a**^

**Site**	**SNP name**	**Position**	**Direction of change**	**Correlated evolution index**	**Major allele**	**Minor allele**	**MAF in** ***pop2***
44	MitoT9900C	9,899	T → C	0.390	T	C	0.016
57	rs41544217	11,812	G → A	0.324	A	G	0.071
72	rs28359178	13,708	G → A	0.318	G	A	0.111

^a^MAF, Mean allele frequency; *pop2*, *BRCA2* mutation carrier.

### Effect quantification

The ALTree method is able to detect an association, but cannot to quantify the associated effect. We estimated the risk of breast cancer for individuals with the T1a1 haplogroup compared with individuals with another T subclade haplogroup in the population of *BRCA2* mutation carriers using a more classical statistical method, a weighted Cox regression. We found a breast cancer HR of 0.55 (95% CI, 0.34 to 0.88; *P* = 0.014). We also tested haplogroup T1a1 and compared it with other T* haplogroups and the H haplogroup (the main haplogroup in the general population), and we found a breast cancer HR of 0.62 (95% CI, 0.40 to 0.95; *P* = 0.03).

## Discussion

We employed an original phylogenetic analytic method, coupled with more classical molecular epidemiologic analyses, to detect mitochondrial haplogroups differentially enriched for affected *BRCA1/2* mutation carriers. We successfully inferred haplogroups for more than 90% of individuals in our dataset. After haplogroup imputation, the ALTree method identified T1a1 in the T clade as differentially enriched in affected *BRCA2* mutation carriers, whereas no enrichment difference was found for *BRCA1* mutation carriers. The T subclade is present in 4% of African populations compared with 11% in Caucasian and Eastern European populations [43]. In our data, the T subclade represented 9.34% of *BRCA1* mutation carriers and 9.30% of *BRCA2* carriers. The ALTree method also identified three potential breast cancer susceptibility loci in mtDNA. The main goals of using the phylogenetic method we used were to improve statistical power by regrouping subclades according to genetic considerations, to limit the number of tests performed and to precisely quantify this number. ALTree identified three SNPs of interest. Whereas the association we observed could possibly be driven by a single SNP, no difference was observed between multivariate and univariate cox models including the three SNPs identified by ALTree (data not shown).

In this study, we investigated to what extent mtDNA variability modified breast cancer risk in individuals carrying pathogenic mutations in *BRCA1/2*. A large proportion of breast cancer heritability still remains unexplained today [44]. Different methods exist to study genomic susceptibility to a disease, such as linkage analyses (which identified the *BRCA1* and *BRCA2* susceptibility genes) or genome-wide association studies (GWASs). However, classical linkage analysis cannot be applied to the haploid mitochondrial genome. Furthermore, commercial GWAS chips available do not adequately capture the majority of mtDNA SNPs. A non-genome-wide and mtDNA-focused approach was required to explore how mtDNA variability influences breast cancer risk. Here we have shown that *BRCA2* mutation carriers with the subclade T1a1 have between 30% and 50% less risk of breast cancer than those with other clades, which, if validated, is a clinically meaningful risk reduction and may influence the choice of risk management strategies.

The association we observed among *BRCA2*, but not *BRCA1*, mutation carriers may reveal a functional alteration that would be specific to mechanisms involving *BRCA2*-related breast cancer. Today, it is established that *BRCA1*- and *BRCA2*-associated breast cancers are not phenotypically identical. These two types of tumors do not harbor the same gene expression profiles or copy number alterations [45]. Breast cancer risk modifiers in *BRCA1/2* mutation carriers have already been identified [46]. However, most of them are specific from one or the other type of mutation carried [47]. It is therefore not surprising that this observation is observed in *BRCA2* mutation carriers only.

Our inability to assign haplogroups to 9% of study participants could have three main explanations. First, given the high mutation rate in the mitochondrial genome, observed combinations of mtDNA SNPs might have appeared relatively recently in the general population, and the corresponding haplotypes might not yet be incorporated into PhyloTree. Second, only one genotyping error could lead to chimeric haplotypes that do not exist, although, given the quality of our genotyping data, this is unlikely. Third, the mitochondrial reference evolutionary tree PhyloTree is based on phylogeny reconstruction by parsimony, and, for some subclades, it might be suboptimal, especially for haplogroups relying on few mitochondrial sequences, as is the case for African haplogroups [48]. In cases of uncertainty, the choice we made to assign the most recent common ancestor to the studied haplotype enabled us to improve statistical power without introducing a bias in the detected association. For the association detected between T, T1* and T2* subclades, the haplogroup inference method used did not bias the counts of affected and unaffected individuals in these subclades. More details are presented in Additional file 6. Furthermore, on the basis of the haplogroup inference with our method of 630 European and Caucasian mtDNA sequences whose haplogroup is known, we successfully assigned the correct main haplogroup and subhaplogroup of 100% of sequences belonging to T, T2* and T1a1* haplogroups.

We quantified the effect corresponding to the detected association by using a more classical approach. We built a weighted Cox regression including inferred haplogroup as an explicative variable. However, the uncertainty in haplogroup inference was not taken into account in this model. Nevertheless, based on haplogroup assignment and regrouping performed in clade T, affected and unaffected counts of individuals in this clade were not biased.

With only 129 loci genotyped over the 16,569 nucleotides composing the mitochondrial genome, we certainly did not explore the full variability of mitochondrial haplotypes. A characterization of individual mitochondrial genomes would require more complete data acquisition methods to be used, such as next-generation sequencing. However, next-generation sequencing has its own limits and challenges, because some regions of the mitochondrial genome are not easily mappable, owing to a high homology with the nuclear genome, among other factors, and important bioinformatics treatment is necessary to overcome sequencing technology biases. Finally, even for a relatively short genome of “only” 16,569 bp, mtDNA sequencing of more than 20,000 individuals would represent a major increase in cost relative to genotyping 129 SNPs.

ALTree identified T9899C, G11812A/rs41544217 and G13708A/rs28359178 as three potential susceptibility sites for the discovered association (see Additional file 7). These three SNPs are located in the coding part of genes *MT-CO3*, *MT-ND4* and *MT-ND5*, respectively. When looking at PhyloTree, T9899C seems to be involved in T1 subclade definition, whereas G13708A and A11812G are involved in T2 subclade definition. Whereas T98899C and G11821/rs41544217 are synonymous SNPs, G10398A leads to a change of amino acid in the final protein (from alanine to threonine). These two synonymous SNPs have never been described in a disease context in the literature. G13708A is also known for being a secondary mutation for Leber’s hereditary optic neuropathy (LHON) and multiple sclerosis [49]. Although the role of secondary mutations in LHON is still controversial, G13708A could be associated with impairment of the respiratory chain in this pathology. G13708A has also been described as a somatic mutation in a breast cancer tumor, whereas it was not present in adjacent normal tissue or in blood leukocytes [50]. A high proportion of mitochondrial somatic tumor-specific variants are also known mtDNA SNPs, which is consistent with the hypothesis that tumor cells are prone to acquire the same mutations that segregate into mtDNA by selective adaptation when humans migrated out of Africa and confronted new environments [51]. Interestingly, the germline variant G13708A has already been shown to be inversely associated with familial breast cancer risk (with the same direction of the association), with a breast cancer odds ratio of 0.47 (95% CI, 0.24 to 0.92) [52]. None of these SNPs have been described in the context of ovarian cancer.

The corrected *P*-value obtained using ALTree in studying clade T is 0.02, which is not highly significant. A replication step should be performed to validate these results. However, it will be difficult to include enough women in this replication step, given the specific profile studied here. In fact, the estimations of *BRCA2* pathogenic mutations in the general population range from 0.068% [5] to 0.69% [53]. T1a1 represents only a small percentage of European haplogroups (from 1% to 2%). The number of women who have this association is therefore low. However, women carrying such mutations are confronted with drastic choices regarding the prevention of breast cancer, notably prophylactic mastectomy or complete hysterectomy. If breast cancer risk is really reduced by a factor of 2 for women with T1a1, this could be an important fact to take into account for breast cancer prevention.

## Conclusions

This study and our results suggest that mitochondrial haplogroup T1a1 may modify the individual breast cancer risk in *BRCA2* mutation carriers. For now, this observation cannot be extended to the general population. Further investigation of the biological mechanism behind the associations we observed may further reinforce the hypothesis that the mitochondrial genome is influential in breast cancer risk, particularly among carriers of *BRCA2* mutations, and, if validated, is of a level to influence cancer risk management choices.

## Additional files

Additional file 1:
**List of ethical committees that approved the access to the data analyzed in this study.**
Additional file 2:
**SNPs selected for downstream analyses.**
Additional file 3:
**Description and results of the procedure used to estimate the accuracy of our haplogroup inference methodology.**
Additional file 4:
**Absolute and relative frequencies of imputed haplogroups by population.** Table containing absolute and relative frequencies of imputed haplogroups for *BRCA1* and *BRCA2* mutation carriers. Additional file 5:
**Correlated evolution index for all non-monomorphic sites observed in short haplotype sequences of subclade T.** Table containing correlated evolution index for all non-monomorphic sites observed in short haplotypes sequences of subclade T. Additional file 6:
**Details of haplogroups inference results for subclade T.**
Additional file 7:
**Methods used to compute coevolution index.**

## Abbreviations

BCAC: Breast Cancer Association Consortium
CI: Confidence interval
CIMBA: Consortium of Investigators of Modifiers of *BRCA1/2*
COGS: Collaborative Oncological Gene-environment Study
DSB: Double-strand break
GWAS: Genome-wide association study
HR: Hazard ratio
LHON: Leber’s hereditary optic neuropathy
MAF: Mean allele frequency
mtDNA: Mitochondrial DNA
mtTree: Phylogenetic tree of the mitochondrial genome
OCAC: Ovarian Cancer Association Consortium
*pop1*: *BRCA1* mutation carrier
*pop2*: *BRCA2* mutation carrier
PRACTICAL: Prostate Cancer Association Group to Investigate Cancer Associated Alterations in the Genome
ROS: Reactive oxygen species
RSRS: Reconstructed Sapiens Reference Sequence
SNP: Single-nucleotide polymorphism
