# Supplementary material for: An original phylogenetic approach identified mitochondrial haplogroup T1a1 as inversely associated with breast cancer risk in BRCA2 mutation carriers
Source: Breast Cancer Res. 2015 Apr 25;17(1):61. doi: 10.1186/s13058-015-0567-2 (PMC4478717; doi:10.1186/s13058-015-0567-2)
Supplement: Additional file 3: — Description and results of the procedure used to estimate the accuracy of our haplogroup inference methodology. [file 13058_2015_567_MOESM3_ESM.docx]

**Additional File 3 : Description and results of the procedure used to estimate the accuracy of our haplogroup inference methodology**

We tested the accuracy of our haplogroup inference method by applying it on public mitochondrial genome sequences of known main haplogroup. 630 full-length mitochondrial sequences, all belonging to frequent Caucasian and European haplogroups - H, I, J, K, U, T, V, W and X - were downloaded from the Human Mitochondrial Genome Database.

(<http://www.genpat.uu.se/mtDB/>, Ingman, M. & Gyllensten, U. mtDB: Human Mitochondrial Genome Database, a resource for population genetics and medical sciences. *Nucleic Acids Res.* **34,** D749–D751 (2006). )

Short haplotypes were reconstructed from these sequences, and haplogroups inferred using our phylogenic analytic method. The method used was unable to assign a haplogroup for 42/630, *i.e.* 6.7% of mitochondrial sequences used (See Supplementary Table S2), whereas it failed for about 9 % of individuals in our experimental dataset. The observed 92 SNPs combinations in these 42 sequences cannot be found in the mitochondrial phylogenetic reference tree PhyloTree v15. These sequences belonged to individuals of different ethnicities: Americans, Europeans and Caucasians (16 individuals), but also Italians (with Sardinians and Sicilians, 8), Finnish (7), Spanish (with Andalusians and Castilians, 5), Armenian (1), Georgian (1), Cherkessk (1), and New Zealander (1). This reinforces the hypothesis that the mitochondrial phylogenic reference tree might be imperfect in some subclades, especially for specific local ethnic groups like Sardinians.

For the 93.3% of sequences that were assigned a haplogroup, we analyzed if the method used successfully inferred the real main haplogroup, and estimate the corresponding accuracy. Results are displayed in Supplementary Table S2. Our method was able to assign a correct haplogroup for 83% of sequences. More precisely, the accuracy reached 100% for sequences belonging to haplogroups I, J, K, T, U, W, X. The accuracy fell to 70% for sequences of H haplogroup, and to 3% for sequences of V haplogroup. For the remaining 30% and 97% of sequences of H and V haplogroups respectively, the inferred haplogroup was HV or HV7. This reveals that with the only 92 SNPs used, our method cannot make the distinction between H and V haplogroups, which are two neighboring subclades in PhyloTree.

We also explored the accuracy of our method to infer subhaplogroup T1, T1a1 and T2. Results are displayed in Supplementary Table S1. Over the 23 T1-haplogroup sequences used, 18 were correctly inferred to T1a1 subhaplogroup. T1a1 has been estimated to represent more than 70% of T1 subclade. The 5 other sequences that belong to T1a3 and T1b subhaplogroups, mainly found Mesopotamia, Anatolia, Egypt, England, Algeria, Greece and India, were inferred T haplogroup by our method. Thus, it is not surprising that the set of SNPs we used in this study that were selected to target European mitochondrial variation does not enable to infer Middle-East haplogroups. The method used here was able to correctly assign T1a1 haplogroup to 100% of sequences belonging to this haplogroup.

Supplementary Table S2: Results of haplogroup inference by using our phylogenetic and analytic method on a set of 623 public mitochondrial genome sequences of known haplogroup

| **Main Haplogroup^a^** | **Initial Number^b^** | **Inferred^c^** | **Inference Rate^d^** | **Success^e^** | **Accuracy (%)^f^** |
| --- | --- | --- | --- | --- | --- |
| H | 155 | 143 | 92% | 100 | 70% |
| I | 18 | 17 | 94% | 17 | 100% |
| J | 74 | 69 | 93% | 69 | 100% |
| K | 50 | 46 | 92% | 46 | 100% |
| T | 72 | 69 | 96% | 69 | 100% |
| U | 143 | 131 | 91% | 131 | 100% |
| V | 64 | 62 | 97% | 2 | 3% |
| W | 42 | 39 | 93% | 39 | 100% |
| X | 12 | 12 | 100% | 12 | 100% |
| *Total* | *630* | *588* | *93%* | *485* | *82%* |
| T1 | 24 | 23 | 96% | 18 | 78% |
| T1a1 | 19 | 18 | 95% | 18 | 100% |
| T2 | 48 | 46 | 96% | 46 | 100% |

^a^ Main haplogroup as indicated on Human Mitochondrial Genome Database (<http://www.genpat.uu.se/mtDB/>)

^b^ Number of full-length sequenced on which haplogroup inference was performed

^c^ Number of sequences for which a haplogroup was inferred with our method

^d^ Number of haplogroup inferred sequences/initial number of sequences

^e^ Number of sequences for which the main or sub-haplogroup has successfully been inferred

^f^ Number of sequences with a correct main or sub-haplogroup inferred / Number of sequences inferred
