# Supplementary material for: An original phylogenetic approach identified mitochondrial haplogroup T1a1 as inversely associated with breast cancer risk in BRCA2 mutation carriers
Source: Breast Cancer Res. 2015 Apr 25;17(1):61. doi: 10.1186/s13058-015-0567-2 (PMC4478717; doi:10.1186/s13058-015-0567-2)
Supplement: Additional file 6: — Details of haplogroups inference results for subclade T. [file 13058_2015_567_MOESM6_ESM.docx]

**ADDITIONAL FILE 6:** **Details of haplogroups inference results for subclade T**

The association we detected with ALTree is located at the first level of Clade T phylogenetic tree (Fig.2). A difference in enrichment for affected and unaffected *BRCA2* mutation carriers was detected between T, T1* and T2* subclades. It is interesting to analyze to what extend the method used to infer individual haplogroup might bias the counts of affected and unaffected individuals in this clade, and potentially lead to a false-positive association.

Supplementary Table S5 presents which haplogroups in clade T are characterized by a same short haplotype, and the most recent common haplogroup they were assigned to.

No T1-haplogroup or T2-haplogropup is misclassified in T-haplogroup individuals counts, since there is only one haplogroup assigned to T. No haplogroup is misclassified between T1*/T2* and T. Counts of affected and unaffected in branches T, T1, and T2 are not biased by the haplogroup inference method used.

**Supplementary Table S5 : Haplogroups by Most Recent Common Ancestor assigned**

| **List of haplogroups characterized by the same short haplotype grouped by Most Recent Ancestor Assigned** | | | | | | | |
| --- | --- | --- | --- | --- | --- | --- | --- |
| **T** | **T1a1** | **T2** | **T2b** | **T2b7a** | **T2c1d2** | **T2d1** | **T2g** |
| T | T1a1 | T2 | T2b | T2b7a | T2c1d2 | T2d1 | T2g |
|  | T1a1_subhap1 | T2_subhap1 | T2b_subhap1 | T2b7a1 |  | T2d1a |  |
|  | T1a1a | T2a1a | T2b_subhap2 |  |  | T2d1b |  |
|  | T1a1a1 | T2a1a_subhap1 | T2b_subhap3 |  |  |  |  |
|  | T1a1b | T2a1a3 | T2b_subhap4 |  |  |  |  |
|  | T1a1b1 | T2a1a5 | T2b1 |  |  |  |  |
|  | T1a1c | T2a1a6 | T2b2 |  |  |  |  |
|  | T1a1d | T2a1b1a | T2b2_subhap1 |  |  |  |  |
|  | T1a1f | T2a1b1a1a | T2b2b |  |  |  |  |
|  | T1a1g | T2a1b2 | T2b2b1 |  |  |  |  |
|  | T1a1h | T2a1b2a | T2b3 |  |  |  |  |
|  | T1a1i | T2a1b2b | T2b3_subhap1 |  |  |  |  |
|  | T1a1j | T2a2 | T2b3a |  |  |  |  |
|  | T1a1k | T2c | T2b3b |  |  |  |  |
|  | T1a1l | T2c1a2 | T2b3c |  |  |  |  |
|  | T1a1 | T2c1c | T2b3d |  |  |  |  |
|  |  | T2c1d | T2b4 |  |  |  |  |
|  |  | T2c1e | T2b4_subhap1 |  |  |  |  |
|  |  | T2c1f | T2b4a |  |  |  |  |
|  |  | T2d | T2b4b |  |  |  |  |
|  |  | T2d2 | T2b4c |  |  |  |  |
|  |  | T2e | T2b4d |  |  |  |  |
|  |  | T2e2 | T2b4f |  |  |  |  |
|  |  | T2e5 | T2b4g |  |  |  |  |
|  |  | T2e6 | T2b4h |  |  |  |  |
|  |  | T2f | T2b5 |  |  |  |  |
|  |  | T2f1 | T2b5a1 |  |  |  |  |
|  |  | T2f1a1 | T2b6 |  |  |  |  |
|  |  | T2f4 | T2b6_subhap1 |  |  |  |  |
|  |  | T2h | T2b6b |  |  |  |  |
|  |  | T2i | T2b7 |  |  |  |  |
|  |  | T2i1 | T2b8 |  |  |  |  |
|  |  | T2j1 | T2b9 |  |  |  |  |
|  |  |  | T2b11 |  |  |  |  |
|  |  |  | T2b13 |  |  |  |  |
|  |  |  | T2b13a |  |  |  |  |
|  |  |  | T2b13b |  |  |  |  |
|  |  |  | T2b15 |  |  |  |  |
|  |  |  | T2b16 |  |  |  |  |
|  |  |  | T2b17 |  |  |  |  |
|  |  |  | T2b17a |  |  |  |  |
|  |  |  | T2b19 |  |  |  |  |
|  |  |  | T2b19b |  |  |  |  |
|  |  |  | T2b21 |  |  |  |  |
|  |  |  | T2b21a |  |  |  |  |
|  |  |  | T2b21b |  |  |  |  |
|  |  |  | T2b22 |  |  |  |  |
|  |  |  | T2b23 |  |  |  |  |
|  |  |  | T2b23a |  |  |  |  |
|  |  |  | T2b24 |  |  |  |  |
|  |  |  | T2b24a |  |  |  |  |
|  |  |  | T2b25 |  |  |  |  |
|  |  |  | T2b28 |  |  |  |  |
|  |  |  | T2b29 |  |  |  |  |
|  |  |  | T2b |  |  |  |  |
|  |  |  | T2b_subhap1 |  |  |  |  |
