# Supplementary material for: An original phylogenetic approach identified mitochondrial haplogroup T1a1 as inversely associated with breast cancer risk in BRCA2 mutation carriers
Source: Breast Cancer Res. 2015 Apr 25;17(1):61. doi: 10.1186/s13058-015-0567-2 (PMC4478717; doi:10.1186/s13058-015-0567-2)
Supplement: Additional file 7: — Methods used to compute coevolution index. [file 13058_2015_567_MOESM7_ESM.docx]

**Additional File 7: Coevolution Index computation**

*From Bardel, Danjean, Hugot, Darlu and Genin. On the use of phylogeny to detect disease susceptibility loci. BMC Genetics, 2005*

For a tree t, a new character S is allocated to each haplotype h. The state of S is "0", "1" or "?" depending on the proportion ($p_{h}$) of cases carrying the haplotype h compared to the proportion $p_{0}$ of cases in the whole sample.

- If $p_{h}<p_{0}-\varepsilon.\sqrt{\frac{p_{h}.(1-p_{h})}{n_{h}}}$, S is coded "0" (high number of controls);
- If $p_{h}>p_{0}-\varepsilon.\sqrt{\frac{p_{h}.(1-p_{h})}{n_{h}}}$ , S is coded "1" (high number of cases);
- Else, S is coded "?" (unknown status).

with $n_{h}$ being the number of individuals carrying the haplotype h.

For each site i, let $R_{i,t}^{'}$and $R_{i,t}^{''}$be the observed number of times each transition (0→1 for $R_{i,t}^{'}$ and 1→0 for $R_{i,t}^{''}$) co-mutates with a 0→1 change of the character S on tree t.

Let $E_{i,t}^{'}$and $E_{i,t}^{''}$ be the expected number of co-mutations on tree t under the hypothesis of a random distribution of the mutations on tree t and an equal probability of mutation on each branch :

$$E_{i,t}^{'}=\frac{m_{i,t}^{'}.s_{t}}{b_{t}}$$

where:

- $m_{i,t}^{'}$ (resp. $m_{i,t}^{''}$) is the number of 0→1 (resp. 1→0) transitions of the site i on tree t;
- $s_{t}$ is the number of 0→1 transition of the character S on tree t;
- $b_{t}$ is the number of branches of tree t.

Then, for each site i on tree t, we measure the correlated evolution of the site i and the character S, by defining $V_{i,t}^{'}$ (resp. $V_{i,t}^{''}$) as follow:

$$\left\{ \begin{aligned} V_{i,t}^{'}=0 if E_{i,t}^{'}=0 \\ V_{i,t}^{'}=\frac{R_{i,t}^{'}-E_{i,t}^{'}}{\sqrt{E_{i,t}^{'}}} if E_{i,t}^{'} \neq0 \end{aligned} \right.$$

Finally, $V_{i}$ is defined as the max between $V_{i,t}^{'}$and $V_{i,t}^{''}$. The site or the two sites corresponding to the highest $V_{i}$ are selected as putative susceptibility sites.

**Sensibility of correlation index to** $\boldsymbol{\varepsilon:}$

As shown upper in the text, $\boldsymbol{\varepsilon}$ influences the determination of character S for each haplotype h.

A change of “S=0$\to$1” or “S=1$\to$0” is counted as a transition.

A change of S when one of the state is “?” is not counted as a transition.

If $\boldsymbol{\varepsilon}$ is high, S will be more often defined as ‘?’, so the number of observed transitions will be lower. The highest $\boldsymbol{\varepsilon}$ is, the more stringent the localization step is.

**Interpretation of** $\boldsymbol{V}_{\boldsymbol{i}}\boldsymbol{:}$

A high value of $V_{i}$ indicates that the number of cotransitions of site i and character S is high than expected.

A negative value of $V_{i}$indicates that the number of cotransitions of character S and site i is less than expected : site i is less polymorphic than expected.
